# ‘Real-life experience’: recurrence rate at 3 years with Hexvix^®^ photodynamic diagnosis-assisted TURBT compared with good quality white light TURBT in new NMIBC—a prospective controlled study

**DOI:** 10.1007/s00345-017-2077-6

**Published:** 2017-08-12

**Authors:** Kevin M. Gallagher, Kayleigh Gray, Claire H. Anderson, Hannah Lee, Sarah Stewart, Roland Donat, Paramananthan Mariappan

**Affiliations:** Edinburgh Urological Cancer Group, Department of Urology, Western General Hospital, Crewe Road South, Edinburgh, EH4 2XU United Kingdom

**Keywords:** Bladder cancer, Non-muscle invasive bladder cancer (NMIBC), Transurethral resection of bladder tumour (TURBT), Photodynamic diagnosis (PDD)

## Abstract

**Purpose:**

To compare the recurrence rate at 3 years (RR-3y) for non-muscle invasive bladder cancer (NMIBC) between good quality (GQ) PDD-TURBT and GQWL-TURBT where PDD is used in routine practice for all new tumours.

**Methods:**

All new, consecutive, NMIBC that received “good quality” criteria first TURBT across a university hospital service were prospectively recruited to this study over a 4-year period. Data were prospectively collected on all WL-TURBTs performed in 2007/8 and compared with PDD-TURBT from 2009/10. Only resection meeting strict “good quality criteria” were included from each cohort to control for resection quality, then cases were further matched 1:1 based on demographic and pathological criteria. The primary outcome was overall and risk group-specific recurrence rate at 3 years.

**Results:**

Of 808 patients recruited, 345 had GQ-TURBT for NMIBC and were included. RR-3y was significantly less for GQ-PDD overall [RR-3y: GQ-PDD: 57/146 (39.0%), GQ-WL: 72/135 (53.3%) OR = 0.56 (0.35–0.90) *p* = 0.02] and on a 1:1 matched pair basis [RR GQ-PDD: 29/118 (24.6) vs. 59/118 (50.0) OR 0.33 (0.19–0.57) *p* < 0.001)]. Benefit was most marked in high-risk patients: RR-3y in high-risk patients treated with GQ-PDD was 25/48 (52.1%) vs. 28/35 (80%) for GQ-WL [OR 0.27 (0.10–0.74) *p* = 0.01].

**Conclusion:**

When adopted for all new bladder tumour resections in routine practice, PDD appears to be associated with significantly reduced recurrence rates at 3 years in our “real life” experience, particularly in high-risk patients.

**Electronic supplementary material:**

The online version of this article (doi:10.1007/s00345-017-2077-6) contains supplementary material, which is available to authorized users.

## Introduction

The long-term impact of photodynamic diagnosis (PDD)-assisted trans-urethral resection of bladder tumour (TURBT) on non-muscle invasive bladder cancer (NMIBC) recurrence rates is not known. There are no prospective controlled studies that examine NMIBC recurrence rates after routine use of hexaminolevulinate (HAL)-PDD-TURBT. There are four randomised controlled clinical trials (RCTs) examining recurrence rates with HAL-PDD-TURBT vs. white light (WL) [1–7] but all have some limitations. Only one [6] has shown benefit of PDD beyond 1 year (24 months). There are three “real-life” observational studies of HAL-PDD all of which are within-patient analyses of *detection* rates and do not examine recurrence rates [8–10]. Prospective controlled studies of a new technique in routine use are required to augment RCTs since RCTs may not represent what happens in the real-life setting [11–13].

The only RCT with follow-up beyond 2 years did not show benefit of PDD [3]. This and all but one [7] other trial included new *and* recurrent tumours. No trial had resection quality standardization and only 2/4 gave any mitomycin C [3]. We know that the quality of resection is a critical determinant of NMIBC recurrence [14, 15] and mitomycin C is proven to significantly reduce recurrence rates [16]. Thus, in addition to the need for a prospective study of PDD in routine use, the other limitations of the RCTs should be addressed. We, therefore, planned this study to provide the first long-term prospective data comparing recurrence rates after routine PDD use vs. WL and to address the limitations in the RCTs. We wanted to demonstrate: (1) Long-term recurrence rates associated with *routine* use of PDD (we have previously demonstrated the benefit of PDD for 3-month recurrence rates in the real-life setting [17]). (2) Long-term recurrence rates with PDD and WL, where *only new* tumours are included and (3) a comparison between PDD and WL where the *quality* of the initial resection can be controlled for and mitomycin C is given to all patients.

Therefore, the aim of this study was to compare total and risk group-specific recurrence rates in “good quality” (GQ) PDD-TURBT and “good quality” WL-TURBT when PDD is used in routine practice for *all new* NMIBC across a service.

## Patients and methods

In 2006, based on national guidelines [18], it was planned that Hexvix^®^ PDD would be introduced for all *new* bladder tumour resections in the coming years. In anticipation of this, we planned a prospective study to collect data on all (consecutive patients) new tumour WL resections performed in 2007/2008 (the baseline control data) and continue once PDD was introduced for all new tumour resections in 2009/2010. The attainment of quality indicators, along with all other study data was prospectively documented on the dedicated study proforma (Figure S1). The quality criteria that were aimed for and documented were: (1) resection done on lists by or with close supervision of the consultant; (2) a standardized proforma with cystoscopic bladder mapping used; (3) TURBT performed with the aim to completely resect all visible tumour and attainment of this documented; and (4) detrusor muscle was to be obtained and histologically confirmed. Patients were only included in the analysis if they had a first TURBT for new NMIBC and all the above quality criteria were met.

All patients received Mitomycin C within 24 h of TURBT, unless contraindicated. Risk stratification was based on 2002 EAU definitions to facilitate comparison with our series of studies [14, 15, 17]. All patients were discussed by cancer multi-disciplinary team that followed local criteria for chemo/immunotherapy usage, criteria for which did not change over the study. It was local policy that only patients with CIS or multi-focal G3 would receive immediate BCG therapy (provided an early re-TURBT did not demonstrate MIBC). For all others, BCG was deferred until detection of residual disease at re-TURBT or until high-grade recurrence. All high-risk patients had early re-TURBT unless the risks outweighed the benefits. All other patients followed standard cystoscopic surveillance. WL resections were performed on the lists of six experienced consultants and PDD resections on lists of two consultants. Throughout the study period, all TURBT pathology specimens were assessed by the same two specialist, senior uro-pathologists using standardized reporting criteria.

The primary end point was the recurrence rate at 3 years (RR-3y). Recurrence was defined as histologically proven tumour occurring any time after first (deemed to be complete) TURBT including at re-TURBT.

Rate of progression to MIBC and any grade and/or stage progression compared with initial resection was also examined.

To further control for confounder, patients were matched one to one on demographic and pathological criteria. Finally, logistical and Cox regression were performed to determine if observed differences were independent of the surgeon performing the resection.

Patients were excluded from end point analysis (censored) if they had not had a documented recurrence and were lost to follow-up, died or became palliative or unfit.

Mean recurrence-free duration was assessed by Kaplan–Meir analysis and log-rank test. Snapshot recurrence and progression rates were compared by Chi-squared test and matched pair analysis by McNemar’s test. Medians (25th, 75th) were compared by Mann–Whitney *U* test. Statistical analysis was performed using SPSS v21.

## Results

Of 808 patients screened for inclusion, 554/808 (68.5%) had NMIBC, 345/554 (62.3%) of whom had confirmed “good quality” resections and were included (Table 1). After censoring of patients who had not had a documented recurrence and who did not achieve 3 years of follow-up, 135/153 (88.2%) WL and 146/192 (76.0%) PDD patients were assessed for recurrence at 3 years (Figure S2).

**Table 1:** Study population break down

All patients			
	WL-TURBT	PDD-TURBT	*p* value
Screened	438	370	
Median age (25, 75)	71.9 (64.4–80.1)	72.1 (62.2–79.8)	0.30
NMIBC at first TURBT	296 (66.8)	258 (69.7)	0.51
Good quality TURBT for NMIBC (included)	153/296 (51.7)	192/258 (74.4)	<0.001
Risk groups			
Low (G1/G2 Ta, single and <3 cm)	65 (43.0)	57 (29.7)	0.01
Intermediate (G1/G2, Ta/T1, ≥3 cm or multiple and <3 cm)	45 (29.8)	71 (37.0)	0.14
High (high risk: G3, Ta/T1 including cis)	41 (27.2)	64 (33.3)	0.19
CIS at first resection	6/153 (3.9)	10/192 (5.2)	0.57
T1	27/153 (17.6)	45/192 (23.4)	0.19
Unifocal	113/153 (73.9)	98/192 (51.0)	<0.001
>3 tumours	12/157 (7.8)	59/192 (30.7)	<0.001
Re-TURBT in high-risk patients	34/41 (82.9)	55/64 (85.9)	0.95
Primary BCG in high-risk patients^a^	11/41 (26.8)	13/64 (20.3)	0.45
Follow-up			
Median follow-up (25, 75)	53.0 (19.9–65.5)	36.6 (22.3–43.2)	<0.001
Completed 3-year follow-up	105/153 (68.6)	127/192 (66.1)	0.65

^a^ Local guidelines for BCG therapy were followed and did not change through the study, these stipulated that BCG would only be given immediately after first TURBT if there was CIS or multifocal G3, otherwise BCG could be deferred until detection of recurrent or residual high-grade disease

### Recurrence rates

**Fig. 1:**
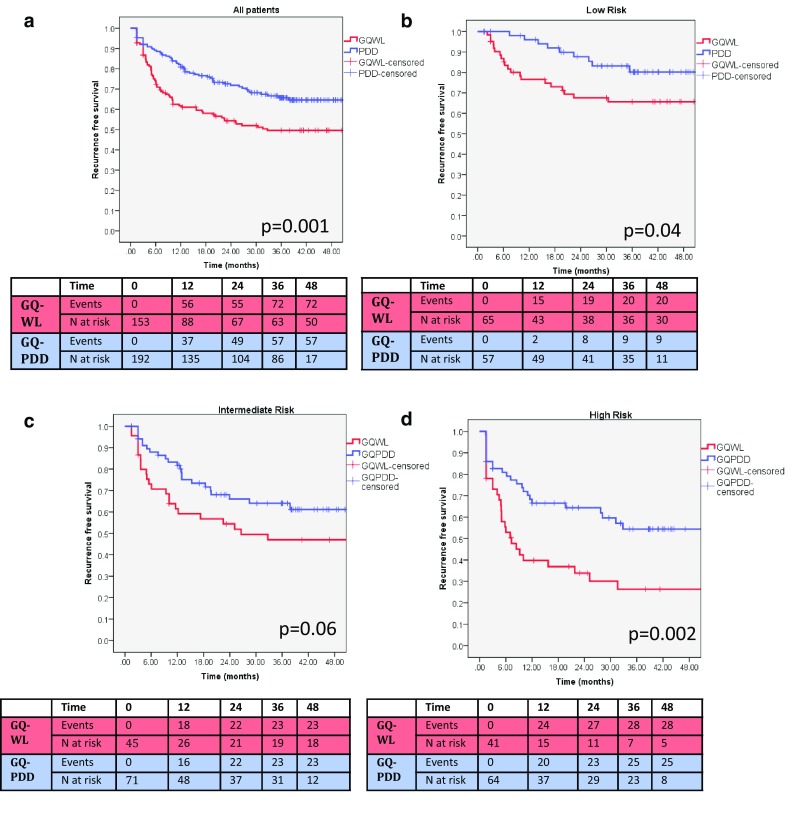
**a** Kaplan–Meir graphs of recurrence-free survival in all patients for GQWL vs. GQPDD (*p* = 0.001, log-rank test). **b**–**d** Kaplan–Meir graphs of recurrence-free survival stratified by risk group

The recurrence rate at 1 year (RR-1y) and RR-3y were significantly less with PDD than WL overall (Table 2A). There was significantly longer mean recurrence-free survival with PDD than WL [PDD: 52.9 (48.4–57.4) vs. WL: 42.4 (36.7–48.1) months *p* = 0.001] (Fig. 1a). There was no significant difference in median time to first recurrence [PDD: 8.7 (3, 21) vs. WL: 5.9 (3, 14.0) months *p* = 0.17].

**Table 2:** Recurrence rates with GQWL-TURBT vs. GQPDD-TURBT

A. All patients						
	1 year			3 years		
	RR-1y (%)	OR (95% CI)	*p* value	RR-3y (%)	OR (95% CI)	*p* value
GQ-WL	56/144 (38.9)	0.43 (0.26–0.71)	<0.001	72/135 (53.3)	0.56 (0.35–0.90)	0.02
GQ-PDD	37/172 (21.5)			57/146 (39.0)		
Low risk						
GQ-WL	15/58 (25.9)	0.11 (0.02–0.54)	0.006	20/56 (35.7)	0.48 (0.19–1.19)	0.11
GQ-PDD	2/51 (3.9)			9/43 (20.9)		
Intermediate risk						
GQ-WL	18/44 (40.9)	0.48 (0.21–1.09)	0.08	23/42 (54.8)	0.61 (0.27–1.38)	0.24
GQ-PDD	16/64 (25.0)			23/54 (42.6)		
High risk						
GQ-WL	24/39 (61.5)	0.34 (0.15–0.79)	0.01	28/35 (80.0)	0.27 (0.10–0.74)	0.01
GQ-PDD	20/57 (35.1)			25/48 (52.1)		

B. Individual subject-matched pair analysis recurrence rate at last follow-up						
RR-3y	WL (%)	PDD (%)	OR (95% CI)	*p* value*		
All	59/118 (50.0)	29/118 (24.6)	0.33 (0.19–0.57)	<0.001		
Low	14/47 (29.8)	7/47 (14.9)	0.41 (0.15–1.14)	0.14		
Intermediate	23/39 (59.0)	10/39 (25.6)	0.24 (0.09–0.63)	0.006		
High	22/32 (68.8)	12/32 (37.5)	0.27 (0.10–0.77)	0.02		

A. 1- and 3-year recurrence rates in all study patients (excluding those who were censored before the end point). B. Individual subject matched-pair analysis of 3-year recurrence rates overall and stratified by risk group. * McNemar’s paired test. Subjects matched on age (±10 years), tumour grade (exact), tumour stage (exact), EAU 2002 risk group (which provides relevant matching for multifocality and tumour size) (exact), length of follow-up (<6 months, 6 months to 1 year, 1–3 years and ≥3 years). 72% of patients in the matched pair analysis had ≥3 years follow-up, 89.8% had >1 year follow-up all had at least one check cystoscopy

Binary logistical regression and Cox regression were performed to determine if the benefit of PDD was independent of the surgeon performing the resection. Surgeon A and B performed 89% of all resections and were compared with the four other surgeons grouped. PDD was associated with significantly reduced recurrence rates at 3 years and significantly better recurrence-free survival independent of surgeon [multivariate Cox HR for PDD vs. WL adjusted for surgeon = 0.57 (0.39–0.83) *p* = 0.003] (Table 3). Considering *only* the two higher volume surgeons, recurrence rate at 3 years was still significantly less with PDD vs. WL [57/146 (39.0) vs. 52/97 (53.0) OR 0.55 (0.33–0.93) *p* = 0.02]. Since there was more multi-focal, intermediate and high-risk disease identified in the PDD group, this adjusted regression in favour of PDD and thus multi-focality and risk group were not included as variables in the regression analysis.

**Table 3:** Binary logistical and Cox regression of GQWL vs. GQPDD and operating surgeon

Regression				
Variable	Binary logistical regression for snapshot 3-year recurrence rate		Cox regression for disease-free survival	
	*p* value	Multi-variate odds ratio (95% CI)	*p* value	Multi-variate hazard ratio (95% CI)
WL (ref)		1.00		1.00
PDD	0.001	0.44 (0.27–0.71)	0.003	0.57 (0.39–0.83)
Surgeon A (ref)		1.00		1.00
Surgeon B	0.94	0.98 (0.52–1.85)	0.86	1.05 (0.64–1.71)
Surgeons (other)	0.83	1.08 (0.52–2.24)	0.84	1.05 (0.63–1.75)

Risk group was not included as a variable in regression since there was more intermediate and high-risk disease identified in the PDD group thus would adjust regression in favour of PDD

### Matched pair analysis

Subjects from WL and PDD groups were matched 1:1 based on age (±10 years), grade (exact), stage (exact), risk group (exact) and follow-up time (<6 months, 6 months to 1 year, >1–3 years and ≥3 years). 236/345 (68.4%) patients were successfully matched into 118 pairs. Matched recurrence rate was significantly less for PDD than WL overall (Table 2B).

### Risk groups

The PDD group was significantly less likely to have unifocal disease declared, significantly more likely to have >3 tumours identified and significantly less likely to have low-risk disease (Table 1).

RR-1y was significantly less in low- and high-risk groups with trend to significance in intermediate risk (Table 2A). This difference in snapshot recurrence was only maintained at 3 years in the high-risk group (Table 2A). Recurrence-free survival was significantly better in low- and high-risk subgroups (Fig. 1b–d) with trend to significance in intermediate risk. In the matched pair analysis, PDD was associated with significantly reduced RR in intermediate and high-risk patients (Table 2B).

### Progression

There was less new high-grade disease or MIBC discovered later in initially low/intermediate-risk disease with PDD compared with WL [PDD: 8/146 (5.5%) vs. WL: 18/135 (13.3%) OR 0.38 (0.16–0.90) *p* = 0.03]. The progression rate to MIBC was 9/135 (6.7%) for WL and 9/146 (6.2%) for PDD. MIBC was found at a median of 19.4 (9.2–27.0) months follow-up.

## Discussion

This study is unique and adds significant data to the current literature. It is the only prospective controlled study, to our knowledge, that examines NMIBC recurrence rates with routine PDD use.

This study benefits from high-quality prospective data collection and the ability to compare resections that are matched for “quality criteria” providing control of confounders potentially inherent in the non-randomised design.

The question of long-term impact of PDD on recurrence rates, particularly in high-risk patients has remained unanswered for some time [19]. PDD is considered as a tool which improves operative quality. Operative quality theoretically impacts short-term recurrence. In this study (despite otherwise identical operative quality), in high-risk GQ-PDD, 48% went 3 years with no recurrence compared with 20% of high-risk GQ-WL.

These findings are at odds with the only other large long-term study of patients receiving HAL-PDD where no difference was shown between WL and PDD. The crucial difference between that study and this is that 60% of patients in that study were *recurrent* bladder cancers (average bladder cancer history of 4.4–5 years) [2, 3]. It is possible that the impact of PDD on long-term recurrence rates is only possible if applied at first presentation. Then a truly complete resection identifying all tumours followed by Mitomycin C installation—may result in durable recurrence-free rates. This hypothesis is supported by the significantly greater number of tumours identified in the PDD group here. Although only documented, fully mapped and fully resected disease was included in this study, it is likely that there were more unseen tumours in the WL group resulting in higher recurrence rate. In contrast to other studies [7, 20], our data does not suggest that additional PDD-identified tumours were CIS (no significant difference in CIS rates in WL vs. PDD). The reason for this is not clear but may be due to the select “good quality” resection population included in this study and relatively low CIS rates.

We accept that we do not offer within-patient analysis of findings with and without PDD. However, we feel that the difference in multifocality between WL and PDD is unlikely to be due to a true difference in multifocality but rather due to increased tumour detection by PDD. Increased tumour detection and correct staging may result in more appropriate subsequent management at the time of the index TURBT [21]. The difference in multifocality between the groups is thus not a confounder but a benefit of PDD and identification of this difference is a benefit of the study design (which may not be possible in the trial setting).

Due to local BCG policy (“Patients and methods” section), our BCG usage appears low [22] but our overall BCG rates (after residual disease or recurrence) are higher (not reported). Only the immediate pre-recurrence BCG reported is relevant to the outcome of this study (recurrence rate). Furthermore, both cohorts were managed with identical policy for BCG instillations.

We accept that this is not a randomized trial. We cannot conclusively determine PDD per-se resulted in the improvements observed. It is possible that PDD acted as a learning tool—this itself may be a benefit. We also accept that the sequential temporal nature of these two cohorts is a limitation to our study. We went to great lengths to control for this: sequential patients from each cohort were all included, patients from each cohort that received the highest possible standard of resection could be identified and compared, and further matched one to one based on demographic, risk group, and pathological criteria.

Finally, to be able to recommend routine adoption of PDD for all new tumours, cost–benefit analysis will be required.

## Conclusion

The adoption of PDD for all new bladder tumour resections in routine practice appears to be associated with significantly reduced recurrence rates at 3 years in our “real-life” experience, particularly in high-risk disease.

## Electronic supplementary material

Below is the link to the electronic supplementary material.

Supplementary material 1 (PDF 27 kb) Figure S1. The study proforma used throughout for prospective data collection

Supplementary material 2 (PDF 419 kb) Figure S2. The flow of patients through the study
