# Supplementary material for: ‘Real-life experience’: recurrence rate at 3 years with Hexvix® photodynamic diagnosis-assisted TURBT compared with good quality white light TURBT in new NMIBC—a prospective controlled study
Source: World J Urol. 2017 Aug 12;35(12):1871–7. doi: 10.1007/s00345-017-2077-6 (PMC5693980; doi:10.1007/s00345-017-2077-6)
Supplement: Supplementary file 1 — Supplementary material 1 (PDF 27 kb) Figure S1. The study proforma used throughout for prospective data collection [file 345_2017_2077_MOESM1_ESM.pdf]

Department of Urology, Western General Hospital, Edinburgh

Bladder cancer operation notes / proforma Ver. 2

Name:	Date:
DOB:	Consultant:
CHI Number:	
Hospital Number:	

Operation: **Anaesthesia:**

Surgeon: **Anaesthetist: Dr.**

Indication: First cystoscopy / new tumour / recurrence / check

Findings (delete or circle accordingly):

Tumour number: 1 2 3 >3

Appearance: papillary / solid / mixed
Red patch

Size of largest tumour (mm):
<5 5-10 10-30 >30

Site(s):
R UO L UO Trigone Bl. neck

posterior wall anterior wall

R lateral wall L lateral wall

Anterior wall Dome Diverticulum

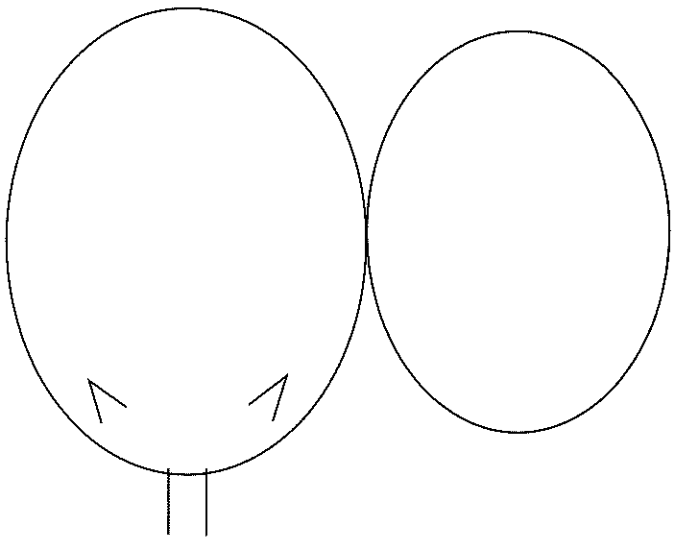

Complete resection: yes / no / not sure / Biopsy and diathermy only

Extra-peritoneal perforation: yes / no / thin wall / Cystoscopy only

EUA: (1) cT1 cT2 cT3 cT4 (2) Bladder mobile: yes / no / not sure

Postoperative Instructions:
(1) Irrigation: yes / no
(2) Intravesical 40mg Mitomycin C within 6 hours: yes / no
(3) TWOC after 24H: yes / no If **no** keep catheter for _____ days
(4) MDT discussion: yes / no If **yes** please complete yellow form
(5) Needs imaging: yes / no If **yes**, please specify:
(6) Other:

Follow up (Please tick):
(1) ☐ GA cystoscopy urgent / in 6 weeks / in 3 months
(2) ☐ GA cystoscopy + Biopsy / diathermy (urgent)
(3) ☐ TURBIT (urgent)
(4) ☐ Flexible cystoscopy in 3 months
(5) ☐ Pending MDT histology and MDT decision

Signature + initials:
