# Supplementary figures and images for: ‘Real-life experience’: recurrence rate at 3 years with Hexvix® photodynamic diagnosis-assisted TURBT compared with good quality white light TURBT in new NMIBC—a prospective controlled study
Source: World J Urol. 2017 Aug 12;35(12):1871–7. doi: 10.1007/s00345-017-2077-6 (PMC5693980; doi:10.1007/s00345-017-2077-6)

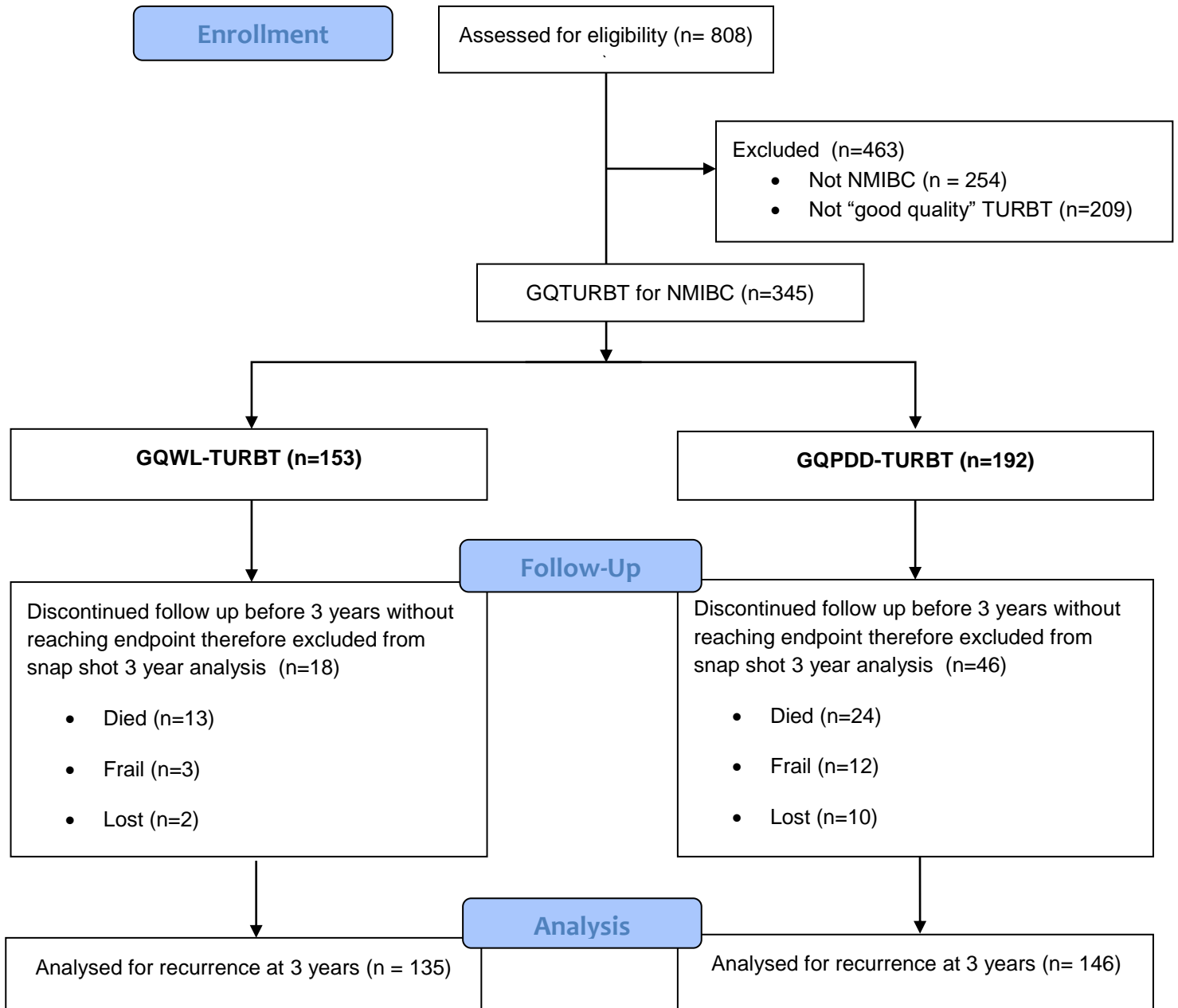

Supplement: Supplementary file 2 — Supplementary material 2 (PDF 419 kb) Figure S2. The flow of patients through the study [file 345_2017_2077_MOESM2_ESM.pdf]
